# Supporting Antenatal Counselling for Anticipated Preterm Births at the Limits of Viability in Non-Tertiary Centres

**DOI:** 10.3390/children12030256

**Published:** 2025-02-20

**Authors:** Alessia Gallipoli, Kyong-Soon Lee, Vibhuti Shah

**Affiliations:** 1 Division of Neonatology, The Hospital for Sick Children, Toronto, ON M5G 1E8, Canada; alessia.gallipoli@unityhealth.to (A.G.); kyong-soon.lee@sickkids.ca (K.-S.L.); 2 Department of Paediatrics, St. Michael’s Hospital, Toronto, ON M5B 1W8, Canada; 3 Department of Paediatrics, Mount Sinai Hospital, 600 University Avenue, Rm 19-231, Toronto, ON M5G 1X5, Canada

**Keywords:** preterm, antenatal counselling, community medicine

## Abstract

**Background:** Presentations of preterm labour at <25 weeks of gestational age (GA) require timely evidence-based counselling and management to optimise outcomes and facilitate informed decisions. In non-tertiary centres, this counselling is often especially challenging. **Objectives**: (1) To develop a tool to support counselling for preterm births at <25 weeks of GA, and (2) to refine and facilitate the utilisation of this tool and develop targeted supports through an understanding of challenges to providing counselling in non-tertiary centres. **Methods**: Perinatal risk factors and local outcome data were incorporated into a counselling tool. Semi-structured virtual interviews were conducted with participants with experience in counselling or receiving care at <25 weeks of GA in non-tertiary centres. Interviewees included transport team members, paediatricians, obstetricians, one family physician, and one parent. Analysis using interpretive description methodology was performed to identify themes in participant practice and experience. **Results**: A risk-based counselling tool was developed, including guidance for counselling discussions. Twenty-one interviews were completed. Practice challenges that were identified included a lack of updated knowledge on practices in tertiary centres, discomfort in providing counselling, variability in counselling content, and a variation in health care provider teams involved in counselling. All providers expressed a desire for further education in this area. **Conclusions**: Support for providers in non-tertiary centres in the counselling of periviable preterm families is much needed. The development of our practice tool targeted for non-tertiary centres provides an important step in this process. The next steps include responding to the expressed need from providers for education and training in the counselling and management of periviable preterm pregnancies.

## 1. Introduction

The presentation of anticipated preterm delivery in non-tertiary centres requires timely management to achieve optimal neonatal outcomes. Preterm infants born outside of a tertiary perinatal centre (“outborn” infants) have worse outcomes compared to their “inborn” counterparts, both in short-term mortality and morbidity and in long-term outcomes [1,2,3]. Despite this, periviable presentations to non-tertiary sites are common, with 37% of initial presentations at <25 weeks of gestational age (GA) in Ontario from 2020 to 2021 being at non-tertiary sites [4]. Additionally, data from the Canadian Neonatal Network (CNN) for the years 2015 to 2021 showed that 14% of infants born at 23 and 24 weeks GA were outborn [4,5].

An important part of management in expected preterm delivery at periviable GAs is the provision of antenatal counselling to facilitate timely management decisions, including the decision for steroid administration and maternal transfer to a tertiary centre [6,7,8,9]. However, despite evidence of the importance of informed antenatal counselling, and the impact of time-sensitive management decisions on neonatal outcomes, there remains variability in how cases of potentially extreme preterm births are managed and how families are counselled at non-tertiary centres [10,11,12,13,14]. This includes variation in management recommendations and survival and outcome data given to families by health care providers (HCPs), especially at the perceived “grey-zone” of viability between 22 and 25 weeks of GA [11,12,13,14]. Counselling at non-tertiary centres is also more likely to reference higher GA cut-offs of viability than those accepted in tertiary centres [11,14]. In a US study, centres without a tertiary Neonatal Intensive Care Unit (NICU) were less likely to provide families with counselling around preterm births, and counselling was less likely to be “risk-based”, meaning the inclusion of prognostic risk factors beyond GA alone, or the use of a standard data source [11]. These counselling inconsistencies are likely multifactorial and have been hypothesised to be impacted by the frequency with which HCPs are faced with extreme preterm deliveries, limitations in the availability of updated resources, and HCP bias [6,7,11]. While variation in antenatal counselling is expected when factors that affect prognosis are considered and incorporated, this variation should not be driven by a lack of HCP knowledge, bias, or institutional barriers [15]. Concerns arise when variability in the counselling provided and resuscitation options presented restrict treatment and impact the ability of families to engage in fully informed and shared decision-making around the imminent delivery and management of their infant [7,9].

Additionally, while many currently available counselling guidelines highlight GA-based outcomes, the incorporation of additional risk factors that influence outcomes can result in more accurate information being provided to families [16]. National guidelines aimed at standardising counselling and management options across regional centres have been developed in the Netherlands, the UK, and Australia [8,15,17,18]. These guidelines include visual aids that incorporate prognostic risk factors beyond GA alone, including the predicted foetal weight and antenatal steroid administration, to aid in prognostication and management advice, along with regional outcome data. The current Canadian Paediatric Society statement highlights the multifactorial elements in this counselling approach [7]; however, a summarised tool or algorithm based on these risk factors is not currently available in Canada.

We aimed to create a risk-based decision-making algorithm with local outcome data to serve as a guide for HCPs during antenatal counselling discussions regarding periviable GAs. Additionally, we sought the perspectives of HCPs working in non-tertiary centres to identify challenges in counselling and provide feedback on the developed tool to facilitate further refinement and targeted support.

## 2. Materials and Methods

### 2.1. Tool Design

A risk-based decision-making algorithm for counselling guidance in cases of preterm labour from 22 to 24 weeks of GA was developed, incorporating known factors that positively and negatively impact outcomes and local outcome data. Best practices around the conduct of communication in antenatal consultation (including setting up an appropriate environment, seeking input on what information parents would find helpful, using judgement and bias-free language, and suggested phrasing for treatment options) were incorporated into the tool design [7,9]. The design of the tool was informed by similarly minded guidelines developed in other jurisdictions internationally [8,17,18], which provide examples of visual-based algorithm designs. Data were obtained from the Better Outcomes Registry and Network (BORN) and the CNN database to provide Ontario data on survival and outcome, including specific information for outborn infants.

### 2.2. Health Care Provider Interviews

The interview and analysis component of this study was completed using interpretive description as a qualitative research approach. Interpretive description focuses on exploring multifactorial experiences to facilitate knowledge translation that focuses on clinically focused outcomes [19,20]. Participants for interviews were identified through purposive sampling to optimise the relevance and impact of perspectives. The sampling method of “key informants” [19] was used to facilitate snowball sampling through the identification of HCPs in positions of leadership, who in turn suggested other relevant participants. Participants were contacted through email to identify interest. Semi-structured group and individual interviews were conducted virtually from January 2023 to April 2023. Interview questions focused on participant experiences in counselling. Providers were also shown a draft of the developed counselling tool for feedback. Participants included four members of the SickKids Acute Care Transport Services, which includes highly trained registered nurses or registered respiratory therapists with specialised training in the counselling, resuscitation, and stabilisation of preterm infants at non-tertiary centres; ten paediatricians; five obstetricians; and a family physician based out of nine non-tertiary hospitals. One parent who was counselled after the presentation of preterm labour at under 25 weeks of GA at a non-tertiary centre prior to transfer to a tertiary centre was also interviewed. Interviews ranged in length from 20 to 60 min and were conducted either virtually using an institutional ZOOM account, or over the telephone. Participants completed an online consent form prior to interview participation. Interviews were recorded and transcribed for analysis. The sample size was guided by the concept of “information power” [21], consistent with the interpretive description guidance that the sample size should be guided by the number of participants needed to produce the knowledge sought [19]. The obtained sample size of 21 participants was felt to achieve this. Research ethics board (REB) approval was obtained through the Clinical Trials Ontario Streamlined Research Ethics Review System, with the REB of the Hospital for Sick Children being the primary research ethics board of approval.

## 3. Results

### 3.1. Counselling Tool

**Figure 1:**
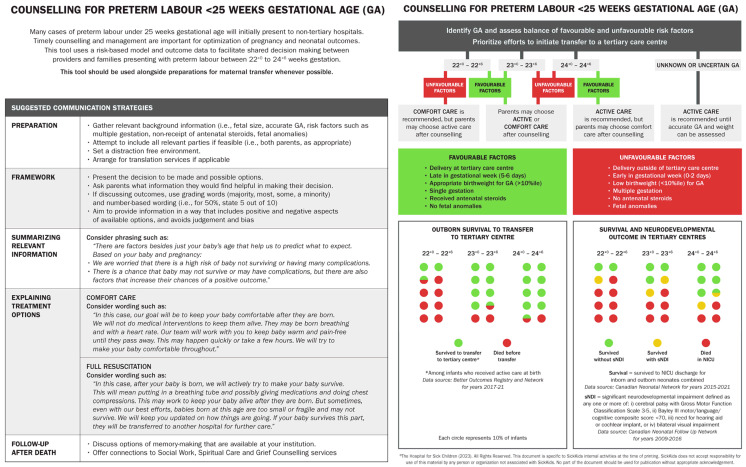
Two-page counselling tool.

The counselling tool was developed as a two-page document (Figure 1). The first page provides a background to the purpose of the tool. The importance of initiating prompt maternal transfer wherever possible is highlighted, as this tool is meant to be used in conjunction with preparation for transfer and is not meant to substitute counselling from a tertiary centre. It also provides a guide to structuring the discussion, including suggestions for specific wording in order to summarise recommendations and explain treatment options to provide additional support for HCPs for whom these conversations may be rare. Information about considerations after a neonatal death is also provided.

The second page of the tool provides an algorithmic approach to providing treatment recommendations based on a balance of positive and negative risk factors. The design asks HCPs to balance the GA and relevant factors to describe treatment options and provide a recommendation where appropriate.

The bottom portion of the tool displays recent Ontario data for neonatal survival and outcome according to GA. One portion provides the proportion of survival during admission to a tertiary NICU site for outborn infants born at 22 to 24 weeks GA in non-tertiary centres who received active resuscitation [defined as the use of ventilation strategies, chest compressions, or resuscitation medications] obtained from available BORN data. This is intended to provide relevant outcome data for cases in which maternal transfer is not possible and parents wish to consider resuscitation. The second portion provides survival and outcome data for infants born at 22 to 24 weeks from CNN data for 2015 to 2021 and is derived from an average of survival over these years from infants admitted to a tertiary NICU site for whom active resuscitation was provided. The CNN data include data from both inborn and outborn infants. Morbidity data were obtained from the 2020 Canadian Neonatal Follow up Network (CNFUN) Annual Report [22]. These data represent outcomes by GA only and do not incorporate risk factors; thus, all risk factor categories are included in each GA data section.

### 3.2. Interview Results

A total of 21 participants with HCP experience in counselling or parents receiving care at <25 weeks of GA in non-tertiary centres were interviewed. This included four members of the Acute Care Transport Services, ten paediatricians, five obstetricians, one family physician, and one parent. The interview content was analysed for the emergence of thematic patterns. From HCP interviews, the following themes featured most significantly: a lack of updated practice knowledge, HCP discomfort in counselling, variability in counselling, inconsistent communication between care teams, challenges in local resources, and a desire for ongoing targeted education and resources. Each thematic pattern is further described below, with examples of illustrative interview quotes provided in Table 1.

#### 3.2.1. Lack of Updated Practice Knowledge

Health care providers often felt that the knowledge available to them and their practice settings around treatment options for periviable gestations was out of date, leading to feelings of disconnect from practice in tertiary centres, which impacted the counselling provided to families. This included specific knowledge around resuscitation cut-offs according to GA and also updated information relating to outcomes and survival for extremely preterm infants.

#### 3.2.2. Health Care Provider Discomfort in Counselling

HCPs expressed feelings of discomfort and inexperience in providing counselling for parents of infants at <25 weeks of GA. These feelings commonly stemmed from feeling unprepared in both knowledge and experience as these patient encounters occur quite infrequently in their settings.

#### 3.2.3. Variability in Counselling

HCPs described experiences of variability in outcomes and treatment options presented to families within the care teams at their centre. This included variability in GA cut-offs where the option for resuscitation was offered. In some cases, the GA cut-off differed among HCPs in the same centre and from the most recently available Canadian Paediatric Society guidelines [7].

#### 3.2.4. Inconsistent Communication Between Care Teams

The level of involvement in counselling of the paediatric and obstetrical teams was found to differ among centres. In some centres, it was expressed that periviable cases, especially at lower GAs, were mainly handled by the obstetrics team, with paediatricians not necessarily becoming involved or being aware of these patients. Other centres adopted approaches for mandatory paediatrician involvement in periviable cases.

#### 3.2.5. Challenges in Local Resources

A recurrent concern from HCPs was the impact of resource limitations in providing care. This included support from tertiary centres for facilitating timely maternal transfer, a lack of local personnel with experience in the resuscitation of these infants, and the incorporation of local factors such as HCP experience with resuscitation and the distance to a tertiary centre for counselling.

#### 3.2.6. Desire for Ongoing Targeted Education and Resources

Each HCP expressed that further education and resources targeting this practice area would be of significant benefit. This included the usefulness of the developed practice tool and the desire for formal educational sessions around this topic.

One interview was completed with a parent with experience presenting in preterm labour to a non-tertiary centre prior to transfer to a tertiary centre. In this discussion, the parent expressed feeling as though they were in a state of waiting prior to being considered eligible for transfer, with communication from HCPs perceived as lacking a sense of hope or a check-in for parental well-being.

## 4. Discussion

The information gathered from interviews with non-tertiary HCPs provided important insights into the challenges they experience during antenatal counselling at potential preterm delivery with periviable GAs. Health care providers lacked updated knowledge from the tertiary centres around practice standards, especially around resuscitation options and outcomes. This lack of knowledge contributed to their discomfort in providing counselling in these cases. There was variability in counselling practices, consistent with findings in previous studies [11,12,13,14]. The creation of our counselling tool helps to address some of these barriers through the provision of a visual algorithmic practice guideline with up-to-date practices and promotes more consistency during counselling. Our tool also includes outcomes that are relevant for non-tertiary settings. For example, survival rates after active resuscitation in non-tertiary centres to allow admission to tertiary centres are available, as well as outcomes after tertiary admission. The inclusion of communication strategies as part of the tool provides guidance for important aspects of the structure and content of these challenging conversations that may be less familiar to HCPs in non-tertiary centres due to the rarity of these events at their centres. The published version of the practice tool has incorporated input and feedback from our interviewees. Feedback from the HCPs on the practice tool was positive and overall they felt that the tool, with its design and outborn-specific data, would be helpful in their practice.

Challenges specific to resource limitations in non-tertiary settings, such as difficulties in timely maternal transfer and a lack of local personnel with neonatal experience, that were expressed in the interviews need to be acknowledged as high priorities. The provincial maternal transfer system needs to be more responsive, timely, and efficient to meet the needs of our non-tertiary centres to expedite maternal transfer whenever possible. Education and training in antenatal counselling and neonatal stablisation should be offered on a regular basis for non-tertiary centres, ideally coordinated through a centralised provincial system.

Input from the one parent interviewed highlighted the importance of communication with families during these scenarios. HCPs may be able to alleviate some of the stresses associated with waiting while being evaluated for eligibility for transfer through timely and ongoing communication.

The strengths of this project include the development of a visual practice tool for counselling for preterm labour at periviable GAs that is targeted for and includes outcome data specific to non-tertiary centres, and is the first such tool developed in Canada. This project also represents the first qualitative examination of barriers and challenges to the provision of counselling for preterm labour in non-tertiary centres.

The next steps will include the dissemination of the tool provincially to facilitate external validation. Dissemination of the tool through educational sessions will include follow-up to gather information on usability in practice and impact from providers and parents, allowing for ongoing evaluation and adjustments. A further report on information gathered through this process is planned. As practices and outcomes for periviable preterm infants continue to evolve, regular review and modification of the tool may be required to continue to meet the needs of our non-tertiary HCPs. Educational sessions will include opportunities for HCPs to identify emerging challenges where ongoing practice support can be focused.

Although our study focused on HCPs mainly located in Southern Ontario, these findings are likely generalisable to the challenges faced in other regions where periviable preterm infants present in non-tertiary centres with varying proximity to an associated tertiary centre and resource availability. This may represent an opportunity for the development of region-specific initiatives, as modifications to the tool may be necessary based on geographical location and centre differences. Although we were able to include only one parent in this stage of the project, ongoing input from parents and the incorporation of parental perspectives in educational sessions will contribute valuable understanding to support families and further educate providers about these challenging situations.

## 5. Conclusions

Support for HCPs in non-tertiary centres in the counselling of periviable preterm mothers is much needed. The development of our practice tool targeted for providers in non-tertiary centres represents an important step in this process. The next steps include responding to the expressed need from HCPs for expanded and targeted education and training in the counselling and stabilisation of periviable preterm pregnancies.

## Figures and Tables

**Table 1:** Examples of provider interview quotes illustrating each practice theme.

Lack of updated practice knowledge	“I think some of the information we sometimes present is a bit outdated” “I spoke to [a colleague], and [they] had no idea that we resuscitated or gave the option of resuscitating 22 weekers”
Health care provider discomfort in counselling	“It’s stressful because it feels almost like, yes, we have some training in this and we might be the best person in the hospital at this given moment, but it’s hard to feel like an expert when you’re not seeing it very often”
Variability in counselling	“I’ve been involved with 24-week twins where until I became aware of them, they were told that’s it” “Our usual counseling at 22 weeks is this is not a viable baby…and that’s what we tell families.”
Inconsistent communication between care teams	“As pediatricians, we very rarely get involved in those. I don’t think they call us … at 22 weeks at all” “Typically, I don’t get too involved with those because they’re really being managed by obstetrics” “Between 22 and 25, paediatrics would always be involved unless there’s going to be a straightforward transfer”
Challenges in local resources	“I think particularly given how far we are from the downtown hospitals, because we’re two hours away.” “Makes it a challenge to … try and adequately counsel them, while at the same time making sure you have enough time to safely get them [to a tertiary centre]”
Desire for ongoing targeted education and resources	“I think having kind of more updated kind of guidelines would be helpful” “If we had just more of a connection with what was going on downtown” “We were looking for a way of standardizing or having some sort of education as how to provide support if these situations come up”

## Data Availability

The original contributions presented in this study are included in the article. Further inquiries can be directed to the corresponding author.
